# ctDNA- and cfDNA-based liquid biopsy in bladder cancer and urothelial carcinoma: bibliometric mapping and cross-database coverage analysis, 2008-2025

**DOI:** 10.3389/fonc.2026.1906152

**Published:** 2026-09-14

**Authors:** Junhua Li, Chenhao Tang, Longfei Yang, Chen Song, Huixian Pan

**Affiliations:** Department of Urology, Hangzhou Third People’s Hospital, Hangzhou, Zhejiang, China

**Keywords:** bibliometrics, bladder cancer, cfDNA, ctDNA, liquid biopsy, molecular residual disease, urothelial carcinoma

## Abstract

**Background:**

Circulating tumour DNA (ctDNA), cell-free DNA (cfDNA) and urinary tumour DNA are increasingly studied in bladder cancer and urothelial carcinoma. We mapped the development, knowledge structure and clinical emphasis of this ctDNA/cfDNA-centred literature and assessed its coverage across three bibliographic databases.

**Methods:**

Web of Science Core Collection (WoSCC), Scopus and PubMed/MEDLINE were searched on July 31, 2026, for records published in 2008-2025. Reviewer-expanded strategies included liquid biopsy, ctDNA/cfDNA, molecular residual disease, molecular relapse, tumour-informed assays, urinary DNA, methylation, TERT and FGFR3. WoSCC was the primary mapping database; Scopus and PubMed/MEDLINE were coverage comparators. Python, VOSviewer and CiteSpace were used for descriptive and network analyses. Newly retrieved WoSCC records underwent duplicate independent screening and full third-reviewer quality control.

**Results:**

The searches retrieved 1,903 WoSCC, 3,201 Scopus and 230 PubMed/MEDLINE records. After WoSCC deduplication and eligibility assessment, 494 records formed the main set and 698 formed a contextual set. The main set received 17,031 citations (median 19; interquartile range 7-42), and annual output reached 82 records in 2025. Scopus covered 456/494 main records and PubMed/MEDLINE covered 93/494. Among 557 newly assessed records, the reviewers initially agreed on 433 (77.7%; Cohen’s kappa, 0.620); all records then underwent third-reviewer full-text quality control.

**Conclusions:**

The expanded search identifies a larger DNA-focused literature than the original strategy and confirms unequal database coverage. Thematic clusters and citation bursts trace a shift from early methylation-marker research towards ctDNA-based molecular residual disease assessment and perioperative treatment stratification. Bibliometric prominence describes research activity, not diagnostic accuracy, predictive validity or treatment benefit.

## 1 Background

Bladder cancer is a common malignancy of the urinary tract, and urothelial carcinoma accounts for most cases. Its management spans non-muscle-invasive, muscle-invasive and metastatic disease and requires repeated surveillance (1, 2). Cystoscopy, imaging, urine cytology and tissue pathology remain central, but their limitations have encouraged investigation of blood- and urine-derived ctDNA and cfDNA (3).

Liquid biopsy has particular relevance in bladder cancer because urine is in direct contact with the tumour bed. Urinary cfDNA, urinary tumour DNA and DNA methylation markers have been reviewed and studied for detection and recurrence surveillance (4–8). Blood-based ctDNA is more often investigated as a marker of systemic tumour burden, molecular residual disease (MRD), relapse and treatment response (9–12). These are complementary clinical settings, although bibliometric prominence alone does not establish diagnostic accuracy or treatment benefit.

Previous reviews have summarised liquid-biopsy technologies and urine biomarkers in urologic malignancies and bladder cancer (4, 13). Recent bibliometric studies have examined bladder-cancer biomarkers broadly rather than a ctDNA/cfDNA-centred corpus (14, 15). The present study therefore focuses on ctDNA, cfDNA and urinary tumour/cell-free DNA across bladder and urothelial carcinoma, uses Web of Science for the main network analysis, and compares record coverage with PubMed/MEDLINE and Scopus. The objectives were to describe publication growth, collaboration patterns, keyword structure, temporal topic evolution and co-cited knowledge bases, and to quantify database overlap and disease-scope sensitivity.

## 2 Materials and methods

### 2.1 Data source and search strategy

This systematic bibliometric review followed PRISMA 2020 reporting principles where applicable (16). WoSCC, Scopus and PubMed/MEDLINE were searched and exported on July 31, 2026. All searches covered January 1, 2008, to December 31, 2025 without language or document-type restrictions. The date boundary was prespecified for comparability with the submitted analysis and was not interpreted as the beginning of urinary DNA biomarker research.

Equivalent reviewer-expanded concepts were implemented with database-specific syntax. Disease terms for bladder cancer, urothelial carcinoma and transitional-cell carcinoma were combined with liquid biopsy, ctDNA/cfDNA, molecular or minimal residual disease, molecular relapse, tumour/tumour-informed assays, general urine-DNA terms and urine-linked methylation, TERT and FGFR3 terms. WoSCC used TS and PY, Scopus used TITLE-ABS-KEY and PUBYEAR, and PubMed/MEDLINE used MeSH and Title/Abstract fields with publication-date limits. Gallbladder terms were excluded in Scopus and PubMed/MEDLINE. Full executable strategies, fields, restrictions, search dates, export counts and source-file SHA-256 values are provided in **Supplementary Methods S1** and the permanent search audit.

The searches retrieved 1,903 WoSCC records, 3,201 Scopus records and 230 PubMed/MEDLINE records. WoSCC was prespecified as the primary database for bibliometric mapping. Scopus and PubMed/MEDLINE were used to assess database coverage rather than as independent clinical validation cohorts.

### 2.2 Parsing, deduplication and eligibility assessment

Raw records were parsed with Python. Extracted fields included authors, title, source journal, document type, author keywords, indexed keywords, abstract, affiliations, DOI, database identifiers, cited references and citation counts. Within-database deduplication used source identifiers, DOI plus normalised title and conflict-safe title-based fallback rules. Ten WoSCC and four Scopus duplicates were removed; PubMed/MEDLINE contained no duplicate PMID. The resulting database sets contained 1,893 WoSCC, 3,197 Scopus and 230 PubMed/MEDLINE records.

Eligibility was assessed from titles, abstracts and author keywords; indexed keywords alone were insufficient. The main category required a substantive bladder/urothelial disease focus together with DNA-based liquid-biopsy evidence. Eligible concepts included ctDNA/cfDNA, urinary tumour DNA, DNA-based MRD or molecular relapse, tumour-informed assays and urine-linked DNA methylation, TERT, FGFR3 or general urinary DNA testing. The contextual category retained non-DNA liquid-biopsy or urinary-biomarker literature for scope auditing. Exclusions included absent disease-DNA intersections, gallbladder false positives, calf thymus DNA, veterinary research, incidental pan-cancer mentions, ineligible publication years and ineligible document types.

After deduplication, 20 WoSCC records were outside the final 2008–2025 publication-year range and 407 had ineligible document types. The remaining 1,466 records entered thematic assessment. Previously audited records were retained as the starting evidence base, while 557 newly retrieved records were supplied to JL and CT in separate suggestion-free files. Each reviewer assessed the same titles, abstracts and author keywords without access to the other reviewer’s decisions or program/AI suggestions.

The reviewers initially agreed on 433/557 records (77.74%; three-category Cohen’s kappa = 0.6195) and disagreed on 124. All 557 records subsequently underwent full-text quality control by a third reviewer; final decisions for this newly assessed group were 71 main, 363 contextual and 123 excluded. The 289 records carried forward from the original WoSCC main corpus were also re-screened independently by two reviewers (initial agreement 252/289, 87.2%; Cohen’s kappa = 0.7352), with disagreements resolved by consensus. A final title-level disease and species audit reclassified four scope-conflicting records (two prostate-cancer studies and two veterinary studies) as excluded. Following the complete screening and reconciliation, 494 records formed the final main set, 698 formed the contextual set, 700 were excluded and one report was not retrieved. These mutually exclusive counts reconcile to all 1,893 deduplicated WoSCC records.

The contextual records were retained only to document the eligibility boundary. They were excluded from annual output, citation, collaboration, keyword, timeline and reference-burst analyses. Record-level classifications, reviewer-specific files, consensus decisions, exclusion reasons, cleaning rules and file fingerprints were retained for audit.

### 2.3 Keyword cleaning and bibliometric analysis

Author keywords and Keywords Plus were cleaned before keyword co-occurrence analysis. Cleaning standardised capitalisation, plurals, hyphenation and abbreviations. Synonymous terms were merged: ctDNA and circulating tumour/tumor DNA variants were mapped to ctDNA; cfDNA and cell-free DNA variants were mapped to cfDNA; and liquid biopsy/biopsies were merged.

Missing-value tokens, non-specific disease terms, non-target cancers, circulating tumour cells, non-DNA components, non-informative indexing fragments and trial-design terms such as multicentre, open-label, single-arm and phase II were removed from the DNA-focused network. The complete merge/remove/retain dictionary is provided in **Supplementary Table 9**.

Python was used to summarise annual output, citations accrued by papers grouped by publication year, document type, sources, contributors, database overlap and sensitivity analyses. VOSviewer 1.6.20 was used for country/region and institution co-authorship networks and keyword co-occurrence and overlay maps (17). Full counting was used; publications with multiple countries or institutions were counted once for each. Association-strength normalisation and the default clustering settings were retained. Countries and institutions required at least five publications. The primary combined-keyword network used a minimum occurrence threshold of eight. Thresholds of 5, 8 and 10 and an author-keyword-only analysis were examined for sensitivity.

CiteSpace 6.4.R2 was used to analyse topic evolution and co-cited knowledge bases (18). The time span was 2008–2025 with one-year slices. Node selection used a Top N% criterion per slice, 10% for keywords and 5% for cited references, with at most 100 nodes per slice, a five-year lookback, retain top K = 5 and link-retaining factor = 2.5. A lower percentage was used for cited references because the cited-reference pool was substantially larger; all other settings were identical. Keyword stopwords and alias merging were applied before keyword analysis and cited references occurring fewer than three times in the 494-record corpus were removed before co-citation network construction (13,344 unique references reduced to 1,833). Pathfinder and pruning of sliced networks were applied; the merged network was not pruned. Clusters were named with log-likelihood ratio labelling. Citation bursts were detected with Kleinberg’s algorithm (gamma = 1.0, two states, minimum duration two years). Cluster quality was assessed with modularity Q and weighted mean silhouette.

### 2.4 PubMed/MEDLINE and Scopus cross-database coverage datasets

Scopus and PubMed/MEDLINE used the same expanded concept blocks as WoSCC, translated into database-specific executable syntax. Records were matched across databases by DOI, non-conflicting PMID and, when identifiers did not conflict, normalised title and year. Publication-stage and same-study representations were collapsed for publication-level overlap calculations.

These datasets were coverage comparators, not independent eligibility or clinical validation cohorts. Scopus and PubMed/MEDLINE coverage of the final WoSCC main set was recorded at publication level. Neither comparator was used for the WoSCC co-citation, reference-burst or CiteSpace analyses.

For raw-set overlap, publication-level harmonisation yielded 1,889 WoSCC, 3,197 Scopus and 230 PubMed/MEDLINE publications. Pairwise Jaccard indices were calculated as the intersection divided by the union. Coverage of the 494-record final WoSCC main set was reported separately as the proportion present in Scopus, PubMed/MEDLINE or either comparator.

### 2.5 Use of 2026 records

To describe the latest research direction, we conducted a separate structured narrative update. The WoSCC records came from the May 20, 2026, search, and PubMed/MEDLINE was searched and exported on May 26, 2026 using the corresponding disease and DNA-liquid-biopsy terms with publication year restricted to 2026 (**Supplementary Methods S1**; **Supplementary Data S10**). The 2026 supplement included 48 WoSCC main-analysis-related records. PubMed/MEDLINE yielded 31 records; after deduplication and the same thematic cleaning, 29 DNA-focused records were retained. Of these 29 records, 23 overlapped with WoSCC, all by DOI, and 6 were PubMed-only. The deduplicated 2026 supplementary evidence therefore included 54 records. These records were used only in the Discussion. They were not included in annual publication analysis, citation analysis, VOSviewer networks, CiteSpace maps or main result tables.

### 2.6 Use of AI-assisted tools

After completion of the bibliometric analysis and Chinese manuscript drafting, ChatGPT and Codex (OpenAI, GPT-5; https://openai.com; accessed July-August 2026) were used to assist with Chinese-to-English translation, language polishing and document formatting. All AI-assisted output was reviewed and edited by the authors. The bibliometric data, references, figures and scientific claims were checked by the authors.

## 3 Results

### 3.1 Study selection and overall dataset characteristics

WoSCC yielded 1,903 records. Deduplication removed 10 records, leaving 1,893 for screening. Twenty records were outside the final publication-year range and 407 had ineligible document types, leaving 1,466 records for thematic assessment. A final title-level scope audit excluded two prostate-cancer and two veterinary records that conflicted with the prespecified eligibility criteria. Final decisions were 494 main, 698 contextual, 700 excluded and one report not retrieved (**Figure 1**).

**Figure 1:**
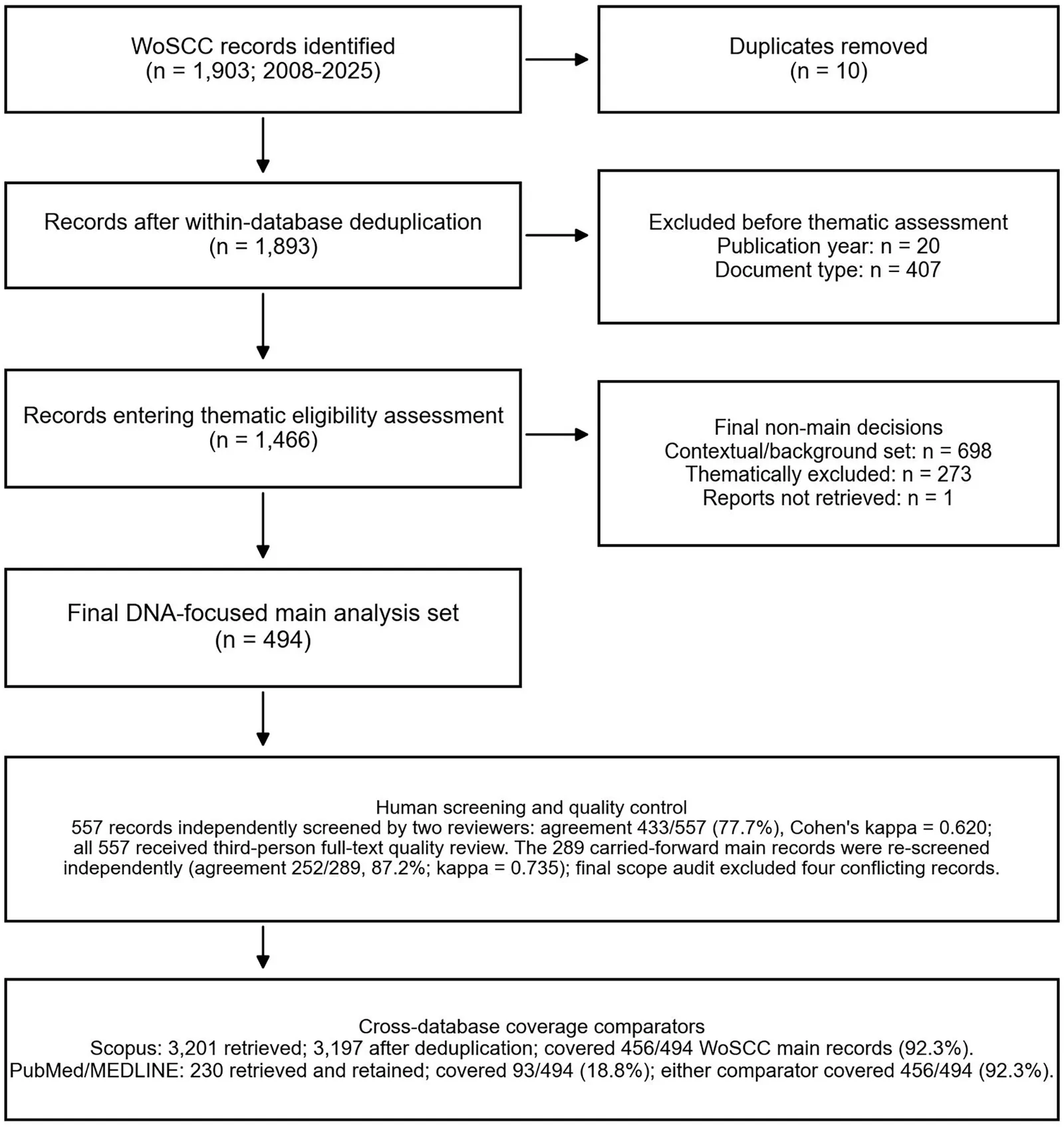
PRISMA-style literature flow and cross-database coverage. WoSCC retrieved 1,903 records and removed 10 within-database duplicates, leaving 1,893 records for screening. Twenty records were excluded by publication year and 407 by document type, leaving 1,466 for thematic assessment. A final scope audit reclassified two prostate-cancer and two veterinary records as excluded. Final mutually exclusive decisions were 494 main, 698 contextual, 700 excluded and one report not retrieved. Among 557 newly assessed records, two reviewers initially agreed on 433 (77.74%; three-category Cohen’s kappa = 0.6195); all 557 then underwent third-reviewer full-text quality control. Scopus retrieved 3,201 records and retained 3,197 after deduplication; PubMed/MEDLINE retrieved and retained 230. Scopus covered 456/494 final WoSCC main records and PubMed/MEDLINE covered 93/494. External databases were coverage comparators rather than independent validation cohorts.

The 494 main records had received 17,031 WoSCC citations by export (mean 34.48; median 19; interquartile range 7-42). Document types were Article (n = 375), Review (n = 118) and Article; Early Access (n = 1) (**Table 1**). The 698 contextual records documented the eligibility boundary and were excluded from the main bibliometric analyses.

**Table 1:** Main-analysis characteristics and bibliometric parameters.

Indicator	Result
Database retrieval	WoSCC, 1,903; Scopus, 3,201; PubMed/MEDLINE, 230
WoSCC after within-database deduplication	1,893 records; 10 duplicates removed
Final WoSCC main dataset	494 records
Contextual dataset	698 records, excluded from main bibliometric analyses
Excluded/not retrieved	700 excluded; 1 report not retrieved
Total Web of Science citations in the main dataset	17,031
Citation distribution per record	Mean 34.48; median 19; interquartile range 7-42
Main document types	Article, 375; Review, 118; Article; Early Access, 1
New-record duplicate screening	557 records; 433 initial agreements; 124 disagreements; Cohen’s kappa, 0.6195; third-reviewer full-text quality control, 557/557
Scopus coverage of the WoSCC main dataset	456/494 records (92.3%)
PubMed/MEDLINE coverage of the WoSCC main dataset	93/494 records (18.8%)
Preparatory keyword threshold audit	Combined thresholds 5/8/10: 108/63/49 nodes; author-keyword thresholds 3/5/8: 80/45/28 nodes
VOSviewer/CiteSpace outputs	Keyword co-occurrence: 62 nodes, 979 links, five clusters (VOSviewer 1.6.20, threshold 8); keyword timeline: 83 nodes, 265 links, six clusters, Q = 0.4166, weighted mean silhouette = 0.7939; co-cited-reference network: 155 nodes, 292 links, eight clusters, Q = 0.6878, weighted mean silhouette = 0.9058 (CiteSpace 6.4.R2)

Citation counts were recorded at the WoSCC export date. Cross-database figures describe bibliographic coverage, not independent validation.

Among the 557 newly retrieved records screened by JL and CT, the reviewers initially agreed on 433 (77.74%; three-category Cohen’s kappa = 0.6195). All 124 disagreements were reconciled, and a third reviewer completed full-text quality control of all 557 records. Final decisions in this group were 71 main, 363 contextual and 123 excluded.

Within the main set, 28 records (5.7%) were title-focused on UTUC and 50 mentioned UTUC in any analysed field. Two titles explicitly focused on non-urothelial bladder-cancer histology. Excluding the 30 unique title-focused UTUC or non-urothelial records left 464 records. The median citation count remained 19, the peak publication year remained 2025, annual ranks were nearly unchanged (Spearman rho = 0.998) and the top-15 keyword Jaccard index was 1.000 (**Supplementary Table 5**).

### 3.2 Annual output and citations grouped by publication year

**Figure 2A** shows annual output and citations accrued by papers grouped by publication year. Annual output was 12 records in 2008 and remained below 20 records per year through 2015.

**Figure 2:**
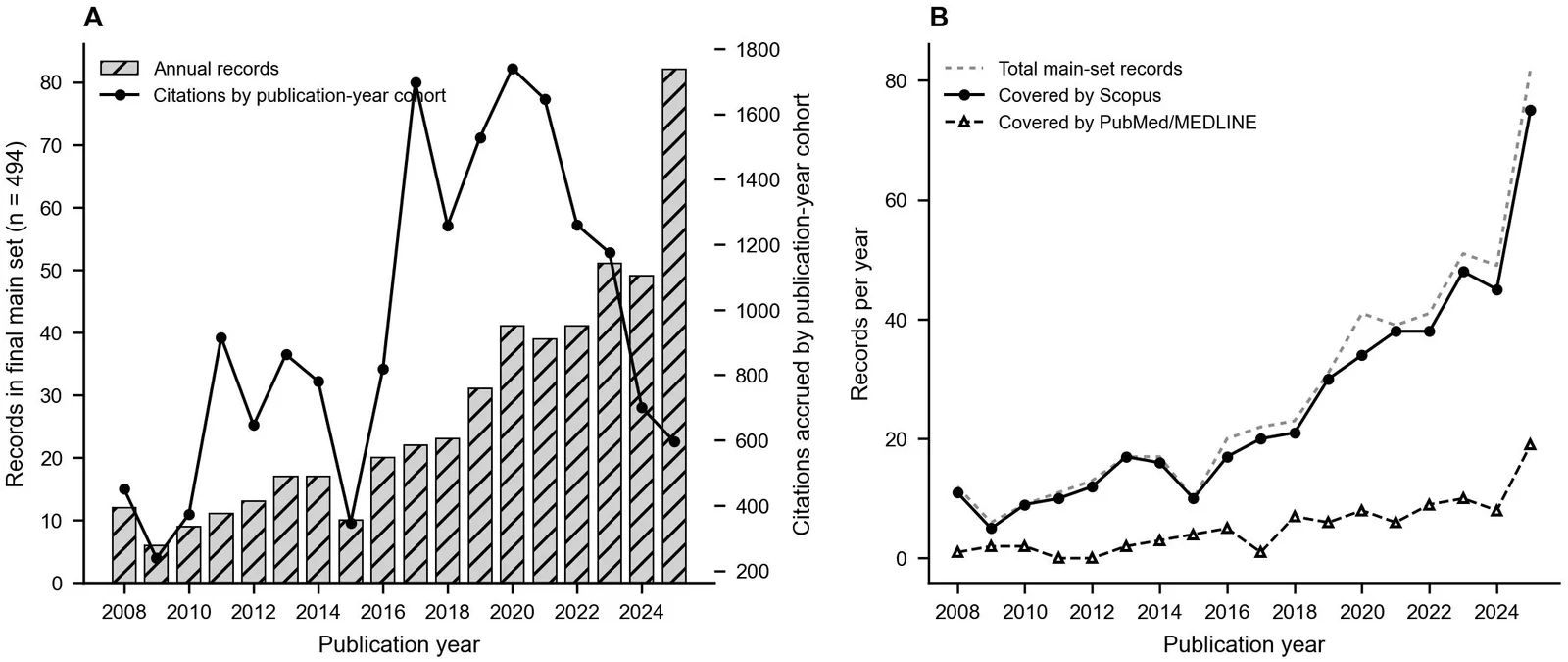
Annual publication output, citation accumulation and cross-database coverage. **(A)** Annual record counts in the 494-record final WoSCC main dataset and citations accrued by papers grouped by publication year as of July 31, 2026. **(B)** Annual numbers of final WoSCC main records covered by Scopus, PubMed/MEDLINE or either comparator. Citation totals are publication-cohort bibliometric measures and do not indicate clinical validity.

Output increased after 2016, with 31 records in 2019, 39 in 2021, 51 in 2023, 49 in 2024 and 82 in 2025. Papers published in 2019, 2021 and 2023 had accrued 1,528, 1,646 and 1,175 citations, respectively, by export. Cohort means, medians, interquartile ranges and top-cited records are provided in **Supplementary Table 6**. These measures describe citation accumulation and were not interpreted as clinical validity or influence.

### 3.3 Cross-database coverage results

After within-database deduplication, the raw retrieval sets comprised 1,893 WoSCC records, 3,197 Scopus records and 230 PubMed/MEDLINE records. Cross-database harmonisation collapsed four additional WoSCC same-study representations, yielding 1,889 publication groups for raw-set overlap calculations.

WoSCC and Scopus shared 1,156 publication groups (union 3,930; Jaccard 0.2941). WoSCC and PubMed/MEDLINE shared 203 (union 1,916; Jaccard 0.1059). Scopus and PubMed/MEDLINE shared 216 (union 3,211; Jaccard 0.0673) (**Table 2**). These metrics describe retrieval-set overlap and not independent eligibility validation.

**Table 2:** Pairwise cross-database overlap.

Database pair	Dataset sizes	Overlap	Unique records	Union	Jaccard index
WoSCC-PubMed/MEDLINE	1,889 and 230	203	1,686 and 27	1,916	0.1059
WoSCC-Scopus	1,889 and 3,197	1,156	733 and 2,041	3,930	0.2941
PubMed/MEDLINE-Scopus	230 and 3,197	216	14 and 2,981	3,211	0.0673

These rows describe the deduplicated raw retrieval sets. DOI and non-conflicting PMID matching preceded standardised-title/year matching. Publication-stage and same-study representations were collapsed to one publication.

Of the 494 final WoSCC main records, Scopus covered 456 (92.3%), PubMed/MEDLINE covered 93 (18.8%) and either comparator covered 456 (92.3%). Coverage patterns were WoSCC plus Scopus (n = 363), all three databases (n = 93) and WoSCC only (n = 38). Annual coverage counts are shown in **Figure 2B** and **Supplementary Table 4**.

Scopus and PubMed/MEDLINE were used only as coverage comparators. They were not treated as independent validation cohorts or used to generate the WoSCC network, timeline or reference-burst results.

### 3.4 Source journals, countries and institutions

The main-analysis records were published in journals covering oncology, urology, molecular diagnostics and translational medicine. The most productive journals were Urologic Oncology-Seminars and Original Investigations (n = 18), International Journal of Molecular Sciences (n = 18), European Urology Oncology (n = 17), Clinical Cancer Research (n = 16), Cancers (n = 16) and European Urology (n = 13). European Urology had the highest citation count among these journals (1,369 citations) (**Table 3**).

**Table 3:** Top productive journals in the main-analysis dataset.

Journal	Publications	Web of science citations
Urologic Oncology-Seminars and Original Investigations	18	503
International Journal of Molecular Sciences	18	474
European Urology Oncology	17	590
Clinical Cancer Research	16	1,173
Cancers	16	354
European Urology	13	1,369
PLOS ONE	11	517
Journal of Urology	12	583
Clinical Epigenetics	10	393
Scientific Reports	10	271

Citation counts were recorded at the WoSCC export date.

Thirty countries or regions met the five-publication threshold and formed six collaboration clusters with 218 links (**Figure 3A**). The United States was the most productive (143 records; 6,603 citations) and had the strongest international connectivity (total link strength 173), followed by China (109 records; 2,811 citations; total link strength 49), Germany (54; 2,632; 87), the Netherlands (46; 2,305; 53), Spain (43; 2,268; 90), Italy (42; 1,633; 72) and England (37; 2,072; 72). Sixty institutions met the threshold and formed nine clusters with 202 links (**Figure 3B**). Aarhus University Hospital (26 records; 2,345 citations; total link strength 30) and Aarhus University (15; 1,010; 31) formed the leading institutional hub, followed by Erasmus MC (17), Shanghai Jiao Tong University (15), the University of Washington (13) and MD Anderson Cancer Center (12).

**Figure 3:**
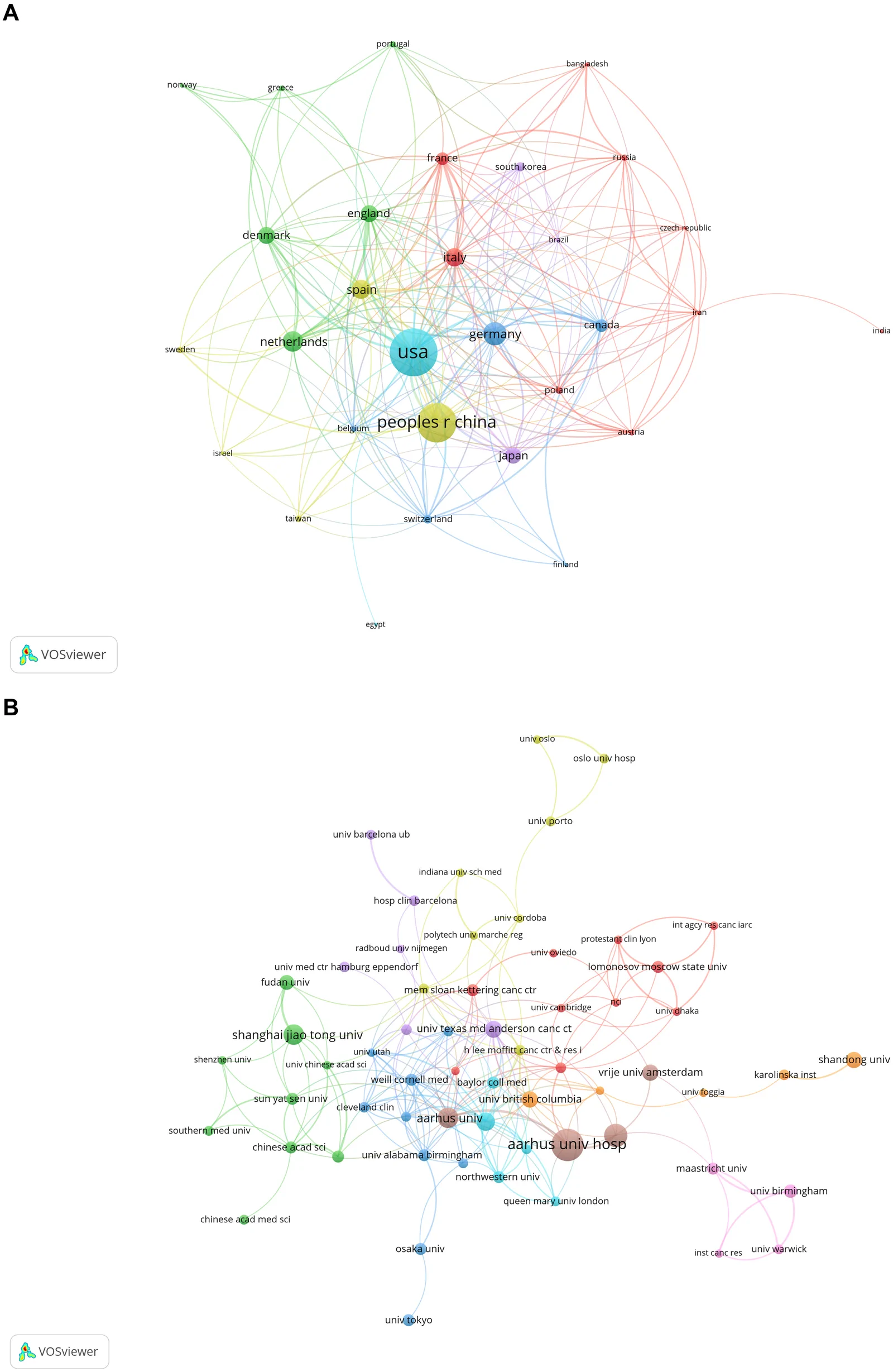
Collaboration networks in the 494-record final WoSCC main set (VOSviewer 1.6.20). **(A)** Countries/regions co-authorship (30 nodes, 218 links, six clusters); **(B)** institutions co-authorship (60 nodes, 202 links, nine clusters). Full counting; publications with multiple countries or institutions were counted once for each; minimum five publications per node; association-strength normalisation; default clustering settings. Node size is proportional to publication count, link thickness to collaboration strength and colours indicate clusters.

### 3.5 Keyword co-occurrence and evolving hotspots

The final VOSviewer keyword analysis of the 494-record dataset used 489 records with combined author or indexed keywords. At the minimum occurrence threshold of eight, the software network contained 62 nodes and 979 links organised into five clusters (**Figure 4**); the reproducible preparatory audit contained 63 nodes and 993 edges because VOSviewer applies its own post-thesaurus node handling. Leading terms included bladder cancer, urothelial carcinoma, liquid biopsy, DNA methylation, ctDNA, cfDNA, surveillance, methylation, urine, cytology, recurrence, mutations, hypermethylation, plasma and urinary biomarkers.

**Figure 4:**
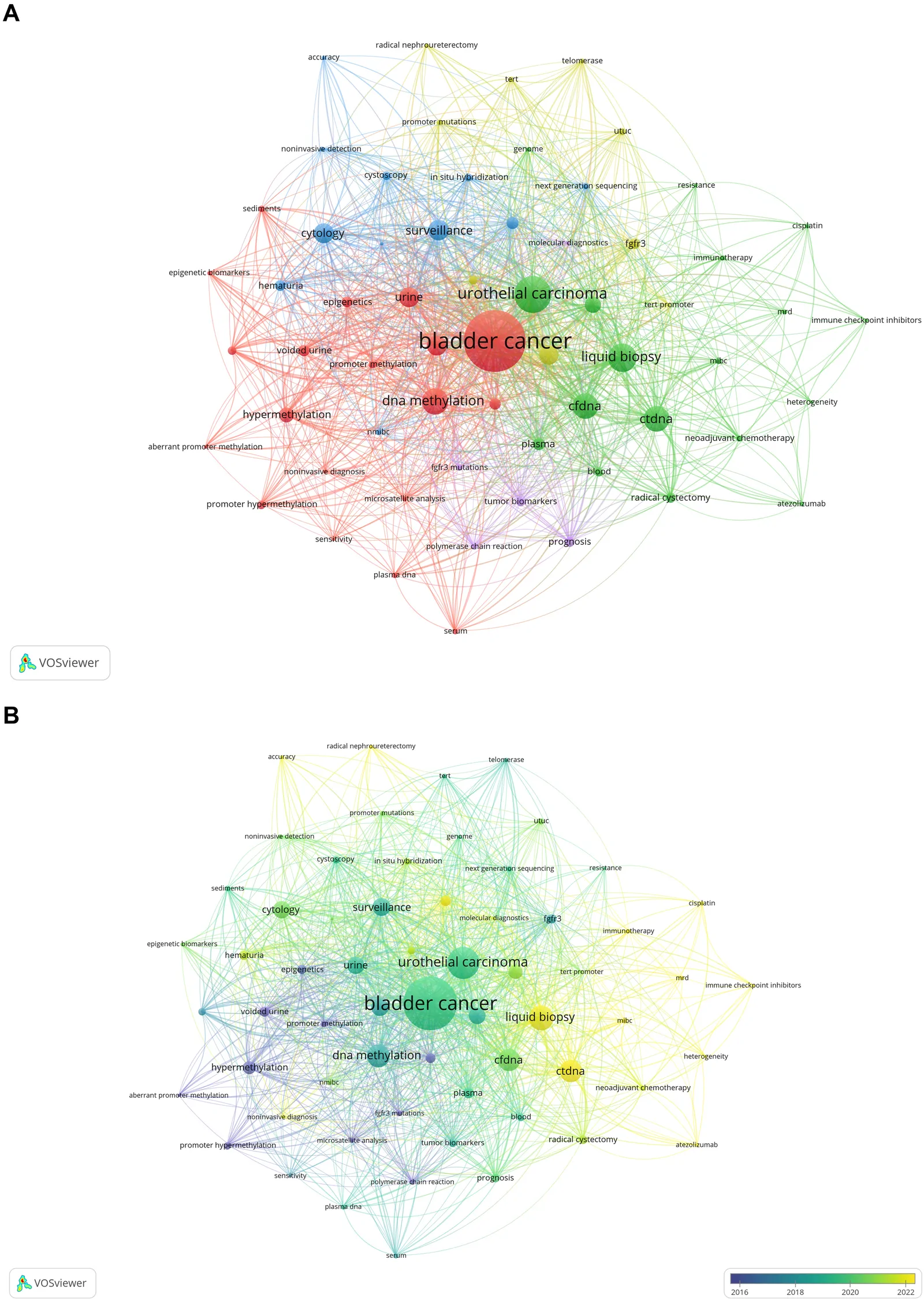
Keyword co-occurrence in the 494-record final WoSCC main set after prespecified synonym harmonisation and removal of non-informative terms (VOSviewer 1.6.20). **(A)** Network visualisation (62 nodes, 979 links, five clusters); **(B)** overlay visualisation in which colour encodes average publication year. Author keywords and Keywords Plus; full counting; minimum occurrence eight; association-strength normalisation. Node size is proportional to occurrence and link thickness to co-occurrence strength. Only software-selected labels are displayed; all eligible nodes remain in the network.

In the overlay visualisation (**Figure 4B**), early foci were methylation-related (aberrant promoter methylation, average publication year 2010.9; promoter hypermethylation, 2012.1; microsatellite analysis, 2015.0), whereas recent terms included MRD (2024.1), immune checkpoint inhibitors (2024.4), MIBC (2023.5) and atezolizumab (2023.1).

Co-occurrence prominence describes research emphasis, not clinical evidence. No diagnostic, prognostic or treatment-effect claim was inferred from node size, link strength, cluster membership or overlay year.

The preparatory threshold audit yielded 108, 63 and 49 combined-keyword nodes at thresholds 5, 8 and 10, with identical top-15 terms. The author-keyword-only analysis included 388 records with usable author keywords and yielded 80, 45 and 28 nodes at thresholds 3, 5 and 8. The author-keyword and combined networks shared the same main DNA-liquid-biopsy themes, while their ordering differed, indicating an influence of database-indexed terms (**Supplementary Table 7**).

### 3.6 Keyword timeline and burst analysis

The CiteSpace keyword network contained 83 nodes and 265 links (modularity Q = 0.4166; weighted mean silhouette = 0.7939), with the largest connected component comprising 81 nodes (97%) (**Figure 5A**; cluster details in **Supplementary Table 15A**). Six clusters were identified: cfDNA (size 20; silhouette 0.826; mean year 2015), machine learning (17; 0.811; 2022), urine (12; 0.742; 2020), mutations (12; 0.823; 2019), DNA methylation (11; 0.801; 2019) and liquid biopsy (9; 0.712; 2019).

**Figure 5:**
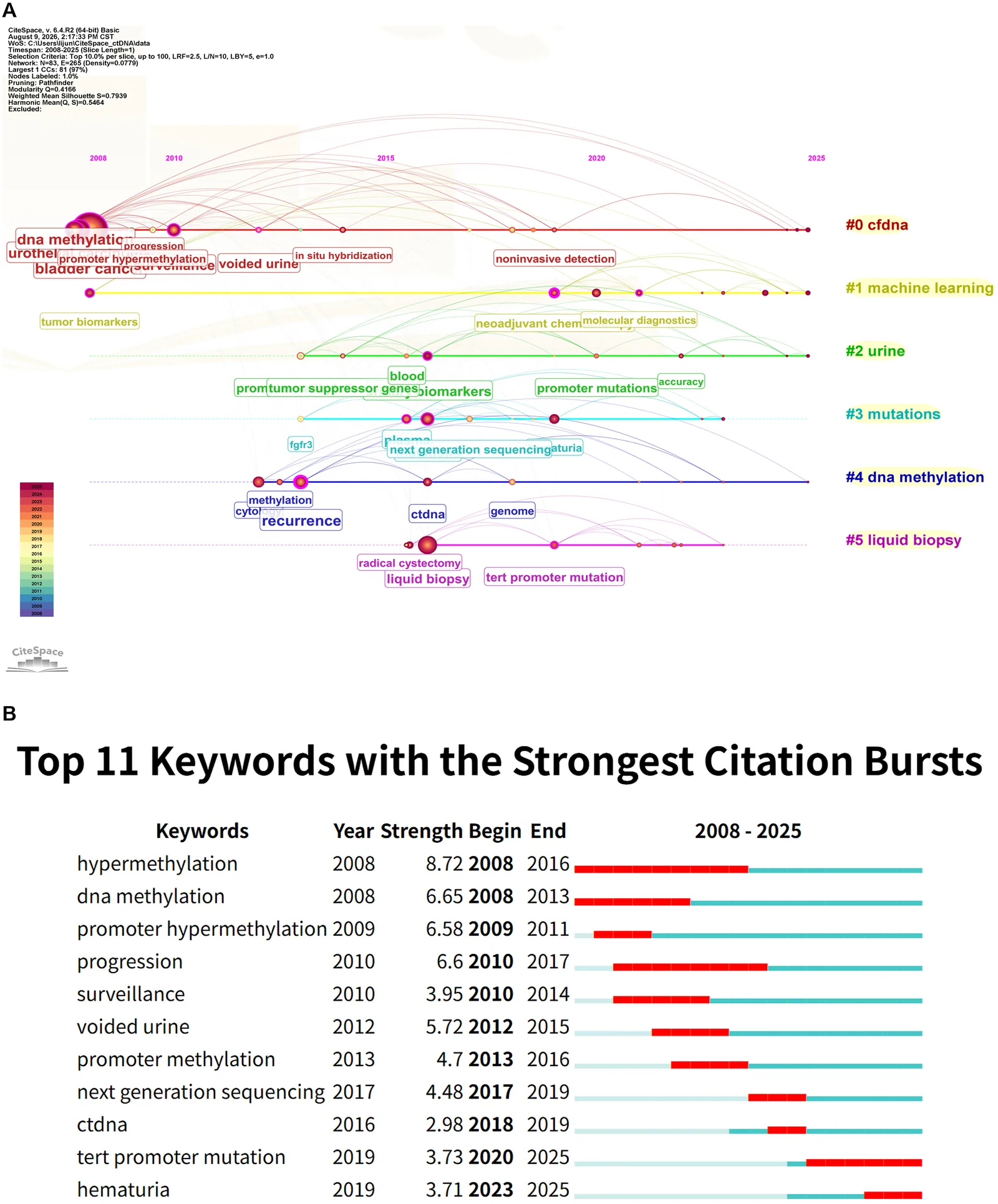
CiteSpace keyword analysis of the 494-record final WoSCC main set (version 6.4.R2; one-year slices, 2008-2025; Top 10% per slice, five-year lookback, retain top K = 5, link-retaining factor 2.5; Pathfinder with pruning of sliced networks; log-likelihood ratio labelling). **(A)** Cluster timeline (83 nodes, 265 links; modularity Q = 0.4166; weighted mean silhouette = 0.7939). The largest connected component contained 81/83 nodes (97%). **(B)** Eleven keywords with citation bursts (Kleinberg’s algorithm; gamma = 1.0; two states; minimum duration two years); red segments mark burst intervals and values give burst strength.

Eleven keywords showed citation bursts (minimum duration two years; **Figure 5B**). The strongest were hypermethylation (strength 8.72; 2008-2016), DNA methylation (6.65; 2008-2013), progression (6.60; 2010-2017), promoter hypermethylation (6.58; 2009-2011), voided urine (5.72; 2012-2015), promoter methylation (4.70; 2013-2016) and next generation sequencing (4.48; 2017-2019). Ongoing bursts were TERT promoter mutation (3.73; 2020-2025) and haematuria (3.71; 2023-2025).

These bursts describe temporary attention in the literature and were not interpreted as evidence of assay performance or treatment benefit.

### 3.7 Reference timeline and burst detection

The co-cited-reference network contained 155 nodes and 292 links (Q = 0.6878; weighted mean silhouette = 0.9058), with the largest connected component comprising 149 nodes (96%) and eight clusters (**Figure 6A**; cluster details in **Supplementary Table 15B**).

**Figure 6:**
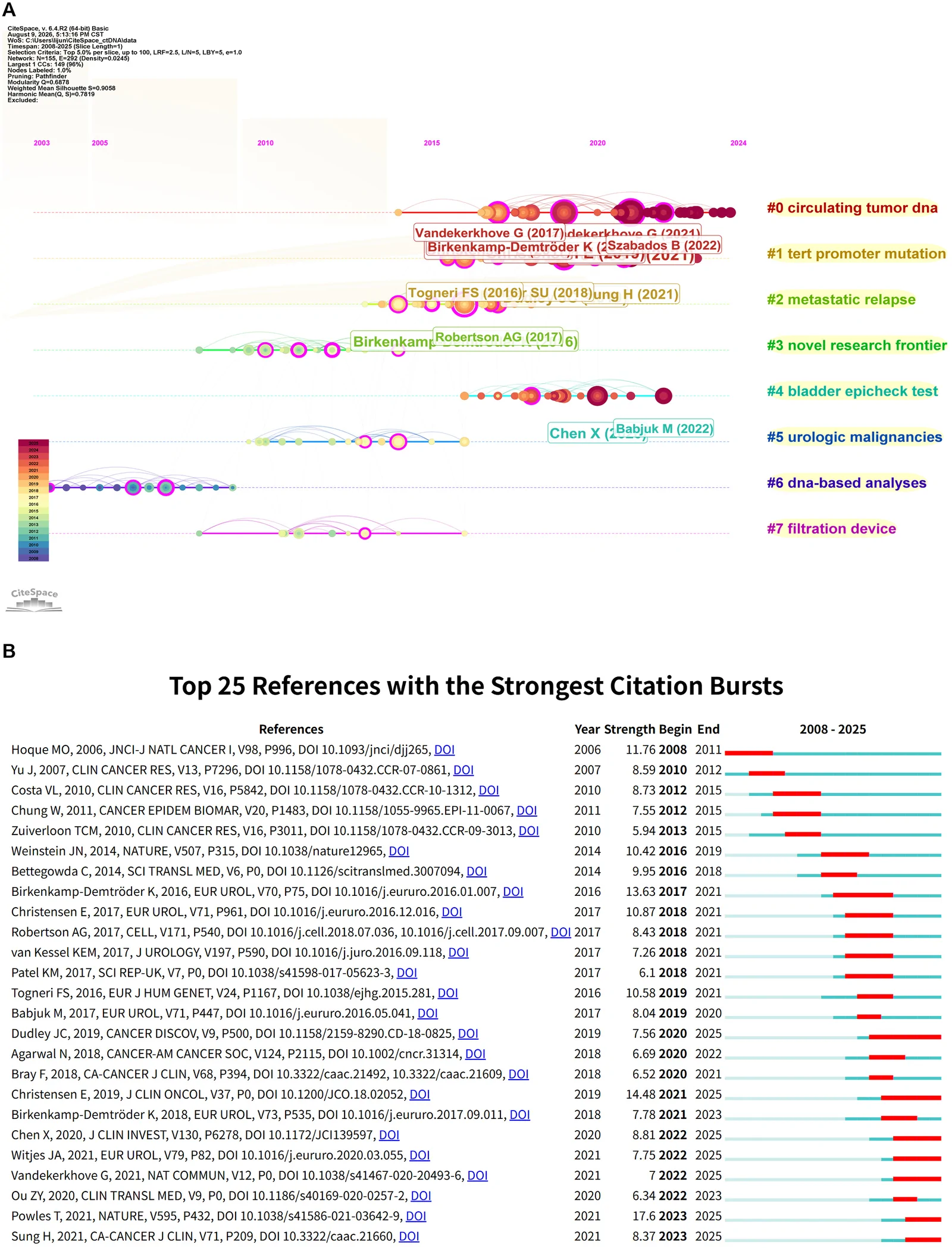
CiteSpace co-cited-reference analysis of the 494-record final WoSCC main set (cited references occurring fewer than three times in the corpus were excluded; 13,344 unique references were reduced to 1,833; Top 5% per slice; other settings as in **Figure 5**). **(A)** Cluster timeline (155 nodes, 292 links; modularity Q = 0.6878; weighted mean silhouette = 0.9058). The largest connected component contained 149/155 nodes (96%). **(B)** Twenty-five references with the strongest citation bursts. Burst strength reflects temporary citation attention, not clinical importance.

Twenty-five references showed citation bursts (**Table 4**; **Figure 6B**). The strongest were Powles et al. (2021; 17.60; 2023-2025), Christensen et al. (2019; 14.48; 2021-2025), Birkenkamp-Demtröder et al. (2016; 13.63; 2017-2021), Hoque et al. (2006; 11.76; 2008-2011) and Christensen et al. (2017; 10.87; 2018-2021). Bursts continuing to 2025 included Dudley 2019, Christensen 2019, Chen 2020, Witjes 2021, Vandekerkhove 2021, Powles 2021 and Sung 2021.

**Table 4:** References with the strongest citation bursts (CiteSpace 6.4.R2; Kleinberg’s algorithm, minimum duration two years).

Reference	Year	Strength	Begin	End
Hoque MO, 2006, JNCI-J Natl Cancer Inst	2006	11.76	2008	2011
Yu J, 2007, Clin Cancer Res	2007	8.59	2010	2012
Costa VL, 2010	2010	8.73	2012	2015
Chung W, 2011	2011	7.55	2012	2015
Zuiverloon TCM, 2010	2010	5.94	2013	2015
Weinstein JN (TCGA), 2014, Nature	2014	10.42	2016	2019
Bettegowda C, 2014, Sci Transl Med	2014	9.95	2016	2018
Birkenkamp-Demtröder K, 2016, Eur Urol	2016	13.63	2017	2021
Christensen E, 2017, Eur Urol	2017	10.87	2018	2021
Robertson AG, 2017, Cell	2017	8.43	2018	2021
van Kessel KEM, 2017	2017	7.26	2018	2021
Patel KM, 2017	2017	6.10	2018	2021
Togneri FS, 2016	2016	10.58	2019	2021
Babjuk M, 2017	2017	8.04	2019	2020
Dudley JC, 2019, Cancer Discov	2019	7.56	2020	2025
Agarwal N, 2018	2018	6.69	2020	2022
Bray F, 2018	2018	6.52	2020	2021
Christensen E, 2019, J Clin Oncol	2019	14.48	2021	2025
Birkenkamp-Demtröder K, 2018	2018	7.78	2021	2023
Chen X, 2020, J Clin Invest	2020	8.81	2022	2025
Witjes JA, 2021, Eur Urol	2021	7.75	2022	2025
Vandekerkhove G, 2021	2021	7.00	2022	2025
Ou ZY, 2020	2020	6.34	2022	2023
Powles T, 2021, Nature	2021	17.60	2023	2025
Sung H, 2021	2021	8.37	2023	2025

Burst detection used the final 494-record WoSCC dataset after removal of references cited fewer than three times in the corpus. Burst strength reflects time-limited citation attention, not clinical importance.

Citation bursts reflect time-limited attention and do not independently establish clinical importance or benefit.

## 4 Discussion

### 4.1 Main findings

This analysis identified 494 DNA-focused bladder and urothelial carcinoma records from 2008 to 2025. Output increased after 2016 and was highest in 2025. The broader retrieval captured earlier methylation, mutation and urine-DNA studies that were under-represented by the submitted ctDNA/cfDNA-centred strategy.

The collaboration structure was led by the United States and China, with a leading institutional hub around Aarhus University. The keyword and cluster structure reproduced the field’s chronological arc: methylation-marker research dominated the early period, ctDNA and sequencing terminology rose after 2016, and MRD- and perioperative-immunotherapy-related terms were the most recent foci. The co-cited knowledge base showed a parallel progression, from early urinary-marker and methylation studies through the TCGA-era genomic characterisation to the perioperative MRD trials. These patterns describe the organisation of the literature and do not imply diagnostic or predictive validity.

### 4.2 Cross-database coverage and topic boundaries

The cross-database analysis showed unequal coverage. Scopus covered 92.3% of the final WoSCC main set, whereas PubMed/MEDLINE covered 18.8%. Pairwise raw-set Jaccard indices were 0.2941 for WoSCC-Scopus, 0.1059 for WoSCC-PubMed/MEDLINE and 0.0673 for Scopus-PubMed/MEDLINE. These values describe database coverage and retrieval-set overlap, not independent eligibility or clinical validation.

The 698 contextual records made the analyte boundary auditable without entering the main maps. Adding MRD, molecular relapse, tumour-informed assays, methylation, TERT, FGFR3 and general urine-DNA terms materially expanded the primary retrieval. The final results should therefore be read as a map of this explicit expanded strategy, not as a census of every bladder-cancer biomarker study.

### 4.3 Urine DNA and plasma ctDNA represent complementary directions

The keyword and cluster structure suggests two complementary research directions. Reviews of the urinary-biomarker landscape, including Matuszczak et al., describe urinary cfDNA, tumour DNA, TERT promoter mutations and DNA methylation assays for detection and recurrence surveillance (4–8). These clinical applications provide context for the bibliometric themes but were not evaluated for diagnostic accuracy in this study.

Tissue genomic studies have established the molecular heterogeneity of urothelial bladder cancer (19, 20). Plasma ctDNA is more often studied in relation to systemic tumour burden, micrometastatic disease and treatment response (9–11, 21–24). Urine and plasma assays should not be assumed to be interchangeable: the literature addresses different specimen biology, disease states and clinical questions.

### 4.4 Treatment stratification has become a central translational theme

Treatment-related terms occupied recent positions in the final outputs. In the keyword overlay, mrd (average publication year 2024.1) and immune checkpoint inhibitors (2024.0) were among the latest terms, and the newest keyword cluster (mean publication year 2023) was centred on machine learning, tumour biomarkers and adjuvant immunotherapy. The strongest ongoing reference burst was the ctDNA-guided adjuvant immunotherapy analysis of Powles et al. (2021; 2022-2025). Perioperative treatment creates a clinical window for MRD testing, whereas dynamic ctDNA has been investigated during systemic treatment for metastatic urothelial carcinoma (9–12, 24). Whether such assays improve outcomes requires prospective, assay-specific evaluation.

MRD was not always among the most frequent individual keywords. This probably reflects heterogeneous terminology. Studies may use minimal residual disease, molecular residual disease, ctDNA positivity or molecular relapse. The importance of MRD should therefore be interpreted through keyword networks, burst references, timeline clusters and clinical study design, rather than a single keyword frequency.

### 4.5 Collaboration patterns and implications for future work

Collaboration was concentrated rather than diffuse. The United States contributed the largest share of main-set records (142/494) and the strongest collaboration links (total link strength 173). China ranked second in output (109 records) but had weaker international connectivity (total link strength 49), and the leading institutional hub was centred on Aarhus University Hospital and Aarhus University.

Future studies from China and other high-incidence regions could strengthen multicentre cohorts, standardised sample handling and cross-regional validation. Such work would improve the generalisability of ctDNA and urinary DNA findings.

### 4.6 Latest developments in 2026

The separate 2026 narrative update comprised 54 deduplicated records: 48 WoSCC records and 6 PubMed-only DNA-focused records. Topics included ctDNA monitoring, perioperative MRD, urinary tumour DNA, multidimensional liquid biopsy and urinary cfDNA methylation sequencing (25, 26). A prospective study compared ctDNA with tumour tissue for guiding FGFR-targeted therapy in metastatic urothelial cancer (27, 28). These records were not included in the 2008–2025 bibliometric analyses.

On May 15, 2026, the US Food and Drug Administration approved atezolizumab and atezolizumab/hyaluronidase for adjuvant treatment after radical cystectomy in patients with ctDNA-MRD-positive muscle-invasive bladder cancer and approved Signatera CDx as the companion diagnostic (29). This regulatory event is clinical context outside the bibliometric analysis and should not be interpreted as validation of all ctDNA/MRD assays.

### 4.7 Limitations

This study has limitations. First, the main maps used WoSCC records. PubMed/MEDLINE and Scopus were coverage comparators rather than co-equal mapping sources, and the Scopus procedure transferred direct WoSCC matches. Records indexed only elsewhere may have been missed.

Second, the expanded strategy was translated across three database interfaces, but field structures and indexing practices differ. Exact retrieval equivalence cannot therefore be assumed. Third, the 557 newly retrieved WoSCC records underwent duplicate independent screening with moderate agreement beyond chance (77.7%; Cohen’s kappa = 0.620); all 557 then underwent full-text quality control by a third reviewer. Although this strengthened the final classification, subjective boundary decisions and differences between title/abstract and full-text information remain possible.

Fourth, indexed keywords and cleaning decisions affect network structure. We therefore provide the dictionary, threshold analyses and author-keyword-only sensitivity, but alternative rules may yield different clusters. Fifth, citations are time-dependent and reflect publication cohorts rather than clinical influence. Finally, bibliometrics describes literature structure; it cannot establish diagnostic accuracy, predictive validity, treatment benefit or specimen complementarity. These questions require prospective assay studies and trials.

## 5 Conclusions

From 2008 to 2025, the expanded search identified 494 DNA-focused bladder and urothelial carcinoma records. Scopus covered most of this WoSCC main set, whereas PubMed/MEDLINE covered a smaller subset. Excluding title-focused UTUC and non-urothelial records did not materially change annual ranks, citation medians or leading keywords. Network clusters and citation bursts traced the field’s evolution from early methylation-marker research, through plasma and urinary ctDNA sequencing, to molecular residual disease assessment and perioperative treatment stratification. Bibliometric patterns describe research activity and cannot establish assay performance or clinical benefit.

## Data Availability

The original contributions presented in the study are included in the article/**Supplementary Material**. Further inquiries can be directed to the corresponding authors. The derived data and reproducibility materials are publicly available in Zenodo at https://doi.org/10.5281/zenodo.21861199.
