# Supplementary material for: ctDNA- and cfDNA-based liquid biopsy in bladder cancer and urothelial carcinoma: bibliometric mapping and cross-database coverage analysis, 2008-2025
Source: Front Oncol. 2026 Sep 14;16:1906152. doi: 10.3389/fonc.2026.1906152 (PMC13616676; doi:10.3389/fonc.2026.1906152)
Supplement: Supplementary file 1 [file DataSheet1.zip › Table_S15b_reference_cluster_summary_CiteSpace.html]

Cluster Summary (8 Clusters by Size)

|  |  |  |  |  |  |  |  |
| --- | --- | --- | --- | --- | --- | --- | --- |
| **Selected** | **ClusterID** | **Size** | **Silhouette** | **mean(Year)** | **Label (LSI)** | **Label (LLR)** | **Label (MI)** |
| true | 0 | 29 | 0.96 | 2020 | circulating tumor dna; urothelial carcinoma; muscle-invasive bladder cancer; bladder cancer; systematic review \| poor prognosis; genitourinary cancer; emerging biomarkers approaches; longitudinal tumor-informed circulating tumor dna status; undergoing radical cystectomy | circulating tumor dna (315.15, 1.0E-4); invasive bladder cancer (97.74, 1.0E-4); circulating tumor dna detection (96.44, 1.0E-4); prognostic biomarker (85.67, 1.0E-4); circulating tumour dna (78.8, 1.0E-4) | ibcn seminar series (2.39); cytological diagnosis |
| true | 1 | 21 | 0.833 | 2019 | urothelial carcinoma; bladder cancer; urinary cell-free dna; upper tract; tert promoter mutation \| urinary cell-free dna-based treatment; narrative review; disease detection; t mutation; emerging bladder cancer biomarker | tert promoter mutation (124.81, 1.0E-4); upper tract (115.64, 1.0E-4); urinary cell-free dna (112.69, 1.0E-4); clinical application (111.45, 1.0E-4); detection technique (59.19, 1.0E-4) | molecular diagnosis (1.83); liquid biopsy biomarke |
| true | 2 | 20 | 0.894 | 2015 | bladder cancer; muscle-invasive bladder cancer; metastatic relapse; non-muscle invasive bladder cancer; circulating tumour dna \| urothelial carcinoma; dna methylation; noninvasive bladder cancer diagnosis; single-cell sequencing-enabled hexokinase; detecting rare malignant cell | metastatic relapse (89.64, 1.0E-4); using urine tumor dna (65.07, 1.0E-4); monitoring treatment response (61.04, 1.0E-4); liquid biopsy analysis (61.04, 1.0E-4); disease detection (57.03, 1.0E-4) | precision therapy (0.67); cytological diagnosis (0 |
| true | 3 | 18 | 0.942 | 2011 | bladder cancer; novel research frontier; molecular marker; gene-based urinary biomarker; dna-based detection \| urine move; tumor suppressor gene; noninvasive diagnosis; novel panel; 2-center prospective study | novel research frontier (64.85, 1.0E-4); gene-based urinary biomarker (56.68, 1.0E-4); size-based enrichment (48.54, 1.0E-4); exfoliated tumor cell (48.54, 1.0E-4); mir-10a gene (40.4, 1.0E-4) | microrna gene (0.07); novel biomarker (0.07); cyto |
| true | 4 | 17 | 0.958 | 2018 | bladder cancer; bladder epicheck test; early detection; urine-based liquid biopsies; sensitive dna methylation marker \| bladder cancer detection; non-muscle invasive bladder cancer; systematic review; recurrence monitoring; urine dna methylation assay | bladder epicheck test (164.63, 1.0E-4); urothelial carcinoma (125.14, 1.0E-4); circulating tumor dna (101.78, 1.0E-4); urine-based liquid biopsies (64.03, 1.0E-4); sensitive dna methylation marker (64.03, 1.0E-4) | cytological diagnosis (1.2); urine methylation tes |
| true | 5 | 17 | 0.775 | 2012 | urologic malignancies; non-blood circulating tumor dna detection; body fluid; nucleic acid-based biomarker; bladder cancer recurrence \| bladder cancer; urinary fgfr3 mutation analysis; prognostic performance; bladder cancer surveillance; prospective multicenter study | urologic malignancies (83.77, 1.0E-4); non-blood circulating tumor dna detection (58.01, 1.0E-4); nucleic acid-based biomarker (50.79, 1.0E-4); body fluid (50.79, 1.0E-4); bladder cancer recurrence (47.09, 1.0E-4) | muscle-invasive urothelial carcinoma (0.09); novel |
| true | 6 | 16 | 0.991 | 2006 | bladder cancer; dna-based analyses; urine sample; small-cell carcinoma; urinary bladder \| bayesian network analysis; urothelial cell carcinoma; methylation urine analysis; multiplexed methylation profile; tumor suppressor gene | dna-based analyses (77.13, 1.0E-4); epigenetic biomarkers gdf15 tmeff2 (77.13, 1.0E-4); urine sample (77.13, 1.0E-4); urinary bladder (61.59, 1.0E-4); small-cell carcinoma (61.59, 1.0E-4) | multiplexed methylation profile (0.09); cytologica |
| true | 7 | 11 | 0.86 | 2011 | bladder cancer; dna-based detection; filtration device; fgfr3 mutation assay; recurrent bladder cancer \| using size-based filtration; microchip electrophoresis; non-invasive genomic diagnostic method; incident nonmuscle; dna-based detection | filtration device (39.85, 1.0E-4); on-site collection storage (39.85, 1.0E-4); dna-based detection (30.99, 1.0E-4); 3-plex methylation assay (30.87, 1.0E-4); fgfr3 mutation assay (30.87, 1.0E-4) | bladder cancer (0.06); urothelial carcinoma (0.03) |
