# Supplementary material for: ctDNA- and cfDNA-based liquid biopsy in bladder cancer and urothelial carcinoma: bibliometric mapping and cross-database coverage analysis, 2008-2025
Source: Front Oncol. 2026 Sep 14;16:1906152. doi: 10.3389/fonc.2026.1906152 (PMC13616676; doi:10.3389/fonc.2026.1906152)
Supplement: Supplementary file 1 [file DataSheet1.zip › Table_S16_reference_bursts_CiteSpace.html]

View Citation Burst History

# Top 25 References with the Strongest Citation Bursts

References | Year | Strength | Begin | End | 2008 - 2025 || Hoque MO, 2006, JNCI-J NATL CANCER I, V98, P996, DOI 10.1093/jnci/djj265, DOI | 2006 | 11.76 | **2008** | 2011 | ▃▃▃▃▂▂▂▂▂▂▂▂▂▂▂▂▂▂ |
| Yu J, 2007, CLIN CANCER RES, V13, P7296, DOI 10.1158/1078-0432.CCR-07-0861, DOI | 2007 | 8.59 | **2010** | 2012 | ▂▂▃▃▃▂▂▂▂▂▂▂▂▂▂▂▂▂ |
| Costa VL, 2010, CLIN CANCER RES, V16, P5842, DOI 10.1158/1078-0432.CCR-10-1312, DOI | 2010 | 8.73 | **2012** | 2015 | ▂▂▂▂▃▃▃▃▂▂▂▂▂▂▂▂▂▂ |
| Chung W, 2011, CANCER EPIDEM BIOMAR, V20, P1483, DOI 10.1158/1055-9965.EPI-11-0067, DOI | 2011 | 7.55 | **2012** | 2015 | ▂▂▂▂▃▃▃▃▂▂▂▂▂▂▂▂▂▂ |
| Zuiverloon TCM, 2010, CLIN CANCER RES, V16, P3011, DOI 10.1158/1078-0432.CCR-09-3013, DOI | 2010 | 5.94 | **2013** | 2015 | ▂▂▂▂▂▃▃▃▂▂▂▂▂▂▂▂▂▂ |
| Weinstein JN, 2014, NATURE, V507, P315, DOI 10.1038/nature12965, DOI | 2014 | 10.42 | **2016** | 2019 | ▂▂▂▂▂▂▂▂▃▃▃▃▂▂▂▂▂▂ |
| Bettegowda C, 2014, SCI TRANSL MED, V6, P0, DOI 10.1126/scitranslmed.3007094, DOI | 2014 | 9.95 | **2016** | 2018 | ▂▂▂▂▂▂▂▂▃▃▃▂▂▂▂▂▂▂ |
| Birkenkamp-Demtröder K, 2016, EUR UROL, V70, P75, DOI 10.1016/j.eururo.2016.01.007, DOI | 2016 | 13.63 | **2017** | 2021 | ▂▂▂▂▂▂▂▂▂▃▃▃▃▃▂▂▂▂ |
| Christensen E, 2017, EUR UROL, V71, P961, DOI 10.1016/j.eururo.2016.12.016, DOI | 2017 | 10.87 | **2018** | 2021 | ▂▂▂▂▂▂▂▂▂▂▃▃▃▃▂▂▂▂ |
| Robertson AG, 2017, CELL, V171, P540, DOI 10.1016/j.cell.2018.07.036, 10.1016/j.cell.2017.09.007, DOI | 2017 | 8.43 | **2018** | 2021 | ▂▂▂▂▂▂▂▂▂▂▃▃▃▃▂▂▂▂ |
| van Kessel KEM, 2017, J UROLOGY, V197, P590, DOI 10.1016/j.juro.2016.09.118, DOI | 2017 | 7.26 | **2018** | 2021 | ▂▂▂▂▂▂▂▂▂▂▃▃▃▃▂▂▂▂ |
| Patel KM, 2017, SCI REP-UK, V7, P0, DOI 10.1038/s41598-017-05623-3, DOI | 2017 | 6.1 | **2018** | 2021 | ▂▂▂▂▂▂▂▂▂▂▃▃▃▃▂▂▂▂ |
| Togneri FS, 2016, EUR J HUM GENET, V24, P1167, DOI 10.1038/ejhg.2015.281, DOI | 2016 | 10.58 | **2019** | 2021 | ▂▂▂▂▂▂▂▂▂▂▂▃▃▃▂▂▂▂ |
| Babjuk M, 2017, EUR UROL, V71, P447, DOI 10.1016/j.eururo.2016.05.041, DOI | 2017 | 8.04 | **2019** | 2020 | ▂▂▂▂▂▂▂▂▂▂▂▃▃▂▂▂▂▂ |
| Dudley JC, 2019, CANCER DISCOV, V9, P500, DOI 10.1158/2159-8290.CD-18-0825, DOI | 2019 | 7.56 | **2020** | 2025 | ▂▂▂▂▂▂▂▂▂▂▂▂▃▃▃▃▃▃ |
| Agarwal N, 2018, CANCER-AM CANCER SOC, V124, P2115, DOI 10.1002/cncr.31314, DOI | 2018 | 6.69 | **2020** | 2022 | ▂▂▂▂▂▂▂▂▂▂▂▂▃▃▃▂▂▂ |
| Bray F, 2018, CA-CANCER J CLIN, V68, P394, DOI 10.3322/caac.21492, 10.3322/caac.21609, DOI | 2018 | 6.52 | **2020** | 2021 | ▂▂▂▂▂▂▂▂▂▂▂▂▃▃▂▂▂▂ |
| Christensen E, 2019, J CLIN ONCOL, V37, P0, DOI 10.1200/JCO.18.02052, DOI | 2019 | 14.48 | **2021** | 2025 | ▂▂▂▂▂▂▂▂▂▂▂▂▂▃▃▃▃▃ |
| Birkenkamp-Demtröder K, 2018, EUR UROL, V73, P535, DOI 10.1016/j.eururo.2017.09.011, DOI | 2018 | 7.78 | **2021** | 2023 | ▂▂▂▂▂▂▂▂▂▂▂▂▂▃▃▃▂▂ |
| Chen X, 2020, J CLIN INVEST, V130, P6278, DOI 10.1172/JCI139597, DOI | 2020 | 8.81 | **2022** | 2025 | ▂▂▂▂▂▂▂▂▂▂▂▂▂▂▃▃▃▃ |
| Witjes JA, 2021, EUR UROL, V79, P82, DOI 10.1016/j.eururo.2020.03.055, DOI | 2021 | 7.75 | **2022** | 2025 | ▂▂▂▂▂▂▂▂▂▂▂▂▂▂▃▃▃▃ |
| Vandekerkhove G, 2021, NAT COMMUN, V12, P0, DOI 10.1038/s41467-020-20493-6, DOI | 2021 | 7 | **2022** | 2025 | ▂▂▂▂▂▂▂▂▂▂▂▂▂▂▃▃▃▃ |
| Ou ZY, 2020, CLIN TRANSL MED, V9, P0, DOI 10.1186/s40169-020-0257-2, DOI | 2020 | 6.34 | **2022** | 2023 | ▂▂▂▂▂▂▂▂▂▂▂▂▂▂▃▃▂▂ |
| Powles T, 2021, NATURE, V595, P432, DOI 10.1038/s41586-021-03642-9, DOI | 2021 | 17.6 | **2023** | 2025 | ▂▂▂▂▂▂▂▂▂▂▂▂▂▂▂▃▃▃ |
| Sung H, 2021, CA-CANCER J CLIN, V71, P209, DOI 10.3322/caac.21660, DOI | 2021 | 8.37 | **2023** | 2025 | ▂▂▂▂▂▂▂▂▂▂▂▂▂▂▂▃▃▃ |
